# Exploring the novel ameliorative effect of date palm pollen extract in inflammatory bowel disease

**DOI:** 10.1007/s10787-026-02370-8

**Published:** 2026-08-24

**Authors:** Bassant E. Samier, Linda K. Mujahed, Shahd Ragab, Nada Badran Nagieb, Aya A. El-fiky, Ayatollah A. El-Shorbagy

**Affiliations:** Zoology and Entomology Department, Faculty of Science, Helwan University, Cairo, Egypt

**Keywords:** Colitis, Anti-inflammation, DPPE, Natural product, IBD

## Abstract

Inflammatory bowel disease (IBD), encompassing Crohn’s disease and ulcerative colitis, is a chronic condition affecting the gastrointestinal tract with elusive causes, raising global health concerns. The date palm (*Phoenix dactylifera* L.), recognized in traditional medicine, presents various bioactive properties such as antioxidant, anti-inflammatory, and antibacterial effects, potentially benefiting gut and vascular health. This study aimed to investigate the therapeutic effects of ethanolic extracts of date palm pollen on IBD in vivo. Fifty male Wister albino rats were divided into a control group (n = 10) and colitis groups (n = 40), with colitis induced by intracolonic acetic acid administration. Following treatment with saline or date palm pollen extract (DPPE) (50, 100, or 200 mg/kg) for 14 days, metrics such as body weight loss, inflammatory markers (TNF-α, IL-6, IL-1β, and NF-κB), oxidative stress indicators (GST, H_2_O_2_, and MDA), colon histopathology, total leukocytes count and their differentiation, and fecal blood were evaluated. Results showed that colitis induction significantly increased leukocyte counts, pro-inflammatory markers, and oxidative stress, along with notable mucosal and submucosal damage. The treatment with DPPE substantially reduced these inflammatory markers and improved colon tissue histology. This research marks the first report of date palm pollen’s ability to ameliorate acetic acid-induced colitis in rats and suggests its potential use as a natural dietary supplement for enhancing intestinal health and reducing inflammation.

*Article*.

## Introduction

Inflammatory bowel disease (IBD), which includes ulcerative colitis (UC) and Crohn’s disease (CD), is a chronic inflammatory disorder of the gastrointestinal tract with an unclear cause. The global incidence of IBD has been steadily increasing, making it a growing public health concern (Ananthakrishnan *et al*. 2020). Multiple factors are believed to contribute to IBD, including genetic predisposition, environmental influences, alterations in the gut microbiome, and immune system imbalances. These factors collectively lead to impaired intestinal barrier function and immune dysregulation (Fumery *et al*. 2021; Desai and Dhoble 2024).

Over the past three decades, there has been a dramatic rise in IBD-related mortality, with global deaths increasing from 21,418 in 1990 to 42,422 in 2021—a 98.07% increase (Lin *et al*. 2024). Despite advances in treatment, there is currently no cure for IBD (Actis *et al*. 2019). Most therapies aim to control inflammation and reduce symptoms using anti-inflammatory drugs such as corticosteroids, immunomodulators, and biologics(Pithadia and Jain 2011; Lichtenstein *et al*. 2018; Actis *et al*. 2019). However, these treatments often result in variable clinical outcomes and may cause serious side effects (Neuman and Nanau 2012).

Laterly, modulating the gut microbiota through dietary interventions has emerged as a promising alternative approach. This strategy may help reduce intestinal inflammation and restore microbiome balance while also supporting nutritional health (Ananthakrishnan *et al*. 2020).

The date palm (*Phoenix dactylifera* L.) particularly its fruits, seeds, and pollen have long been used in traditional medicine and nutrition. Date palm pollen exhibits a range of bioactive properties, in-cluding antioxidant, anti-inflammatory, antibacterial, antihypertensive, antitumor, hepatoprotective, and reproductive health benefits. Studies suggest it may also positively influence gut and vascular health (Bentrad *et al*. 2017; Desai and Dhoble 2024).

The date palm is natural supplements rich contain carbohydrates, proteins, vitamins, enzymes. Furthermore, flavonoids and phenolic acids condenser active volatile unsaturated flavonoids and phenolic compound with highly anti-inflammatory and antioxidant effect these enabling it to eliminate free radicals and be able to prevent oxidative damage for cells by reduce reactive oxygen species such as hydrogen peroxide (H_2_O_2_) (Habib *et al*. 2023). Furthermore, the date palm modulates pro-inflammatory cytokines such as TNF-α and IL-6 play essential role in inflammatory response in vivo (Neurath 2014). Date palm pollen is known for its antioxidant and anti-inflammatory properties, there is a scarcity of studies investigating its effects in experimental colitis models. In particular, its potential role in attenuating colonic inflammation induced by acetic acid has not been sufficiently explored. The current study was designed to evaluate the therapeutic effects of DPPE in a rat model of acetic acid induced colitis. The study focused on assessing changes in body weight, and colonic tissue damage, along with the modulation of inflammatory and oxidative stress biomarkers. Through this experimental approach, the present study aimed to explore the potential of date palm pollen as a natural candidate for the modulate of IBD.

## Materials and methods

### Ethics statement

Animal experiments were approved by the ethics committee of the Faculty of Science Affiliated to Capital University (Helwan University) (Cairo, Egypt) (Approval number: HU-IACUC/AA0330-80). Clinical trial number: not applicable. In addition, all animals received humane care, and the procedures were performed in accordance with the Principles of Laboratory Animal Care.

### Animals

A total of 50 albino male rats weighing 100–120 gm (VACSERA, Cairo, Egypt) were used for the experiments. Rats were maintained at the Animal Experimental Center of the Department of Zoology, Faculty of Science, and kept at a temperature of 22 ± 2 °C with a relative humidity of 55% and a 12 h light and dark cycle. Rats were randomly divided into five groups, namely, the control, colitis, 50, 100, and 200 mg/kg DPPE groups, with ten rats in each group.

### Ethanol extract of date palm pollen

Date palm pollen grains were obtained readily from the Agricultural Research Center, Giza, Egypt. Two hundred grams of powder were extracted using 80% ethanol 1600 mL at room temperature. The extract was filtered to remove particulate matter and centrifuged at 5000 RCF for 30 min. The clear supernatant was evaporated at 40 °C using a rotary evaporator under reduced pressure until completely dry. Then, the dry extract and stock solution obtained were stored in dark glass containers at 4 °C until further use. DPPE was dissolved in distilled water, and the resulting solution was administered orally to rats.

### Induction of colitis

Rats were divided into experimental groups, with colitis induction achieved via intracolonic administration of acetic acid (AA). A polyethylene catheter (PE-60), inserted 8 cm into the rectum under xylezen anesthesia, was used to deliver 1 mL of 4% AA solution. After 30 s, excess fluid was withdrawn, and the colon was flushed with saline (Özdemi̇R *et al*. 2014).

Post-induction, colitis groups received oral injections of saline, DPPE (50 mg/kg/day), DPPE (100 mg/kg/day), or DPPE (200 mg/kg/day) for 14 days. The doses of DPPE (50, 100, 200 mg/kg/day) were selected based on a previous study (Abdelrahim *et al*. 2025). Rats were weighted daily for 14 days.

### Blood in stool

Intestinal bleeding was assessed by evaluating fecal occult blood in rats were detected by semi-quantitative kit (Biodiagnostic, Giza, Egypt). Fecal samples were collected from each animal at predefined time points (Day 7, Day 10, and Day 14 post-induction). The presence of blood in stool was evaluated using a semi-quantitative scoring system, in which higher scores indicated greater severity of intestinal bleeding. Each animal was scored individually within its respective group. The recorded data allowed comparison between the untreated colitis group and the treated groups, as well as evaluation of both dose-dependent and time-dependent effects of the tested compound on intestinal bleeding. This parameter was used as an indicator of mucosal injury and disease severity in the experimental colitis model (Soyturk 2012).

### Collection of blood samples and colonic tissue

On the 14th day, rats were anesthetized for intracardiac blood collection in an EDTA tube for total leukocyte counts and their differentiation. The total leukocyte counts were analyzed 8 h after sample collection, after which colonic tissues were harvested for analysis.

### Method of leukocytes count analysis

Total leukocytes and their differentiation levels were recorded by an automated hematology analyzer (AGRRINA PH-70P, Egypt).

### Histopathological investigation of colon tissue

To report the DPPE effect on colitis-induced rats, a tissue sample from the colon was fixed in neutral formalin (10%). Samples were embedded in the paraffin, sectioned, and stained with hematoxylin and eosin (Miya et al. 2025).

### Measurement of plasma hydrogen peroxide (H_2_O_2_) and glutathione S-transfrase (GST) levels

Plasma levels of H_2_O_2_ and GST were measured using spectrophotometric kits (Biodiagnostic, Giza, Egypt) according to the manufacturer’s instructions.

### Measurement of intestinal malondialdehyde (MDA) level

Intestinal levels of MDA were measured using colorimetric/fluorometric assay kit (Catalog # K739-100, Bio Vision, USA) according to the manufacturer’s instructions.

### Measurement of cytokines by ELISA assay

Colonic levels of TNF-α and IL-6 were measured using a rat ELISA kit (SunLong Biotech, China), and colonic levels of IL-1β and NF-κB were measured using a rat ELISA kit (Catalog E011Ra, BT LAB, China) according to the manufacturer’s instructions.

### Statistical analysis

Experiments were duplicated at least three times. Statistical analysis was performed using GraphPad Prism (version 8.0; GraphPad Software). One-way analysis of variance (ANOVA) was used to compare different treatments in all parameters except fecal blood; two-way ANOVA was used to compare it. The data are represented as mean ± standard deviations (SD); P-values < 0.05 were considered significant.

## Results

### Effect of DPPE on body weight in colitis-induced rats

**Fig. 1:**
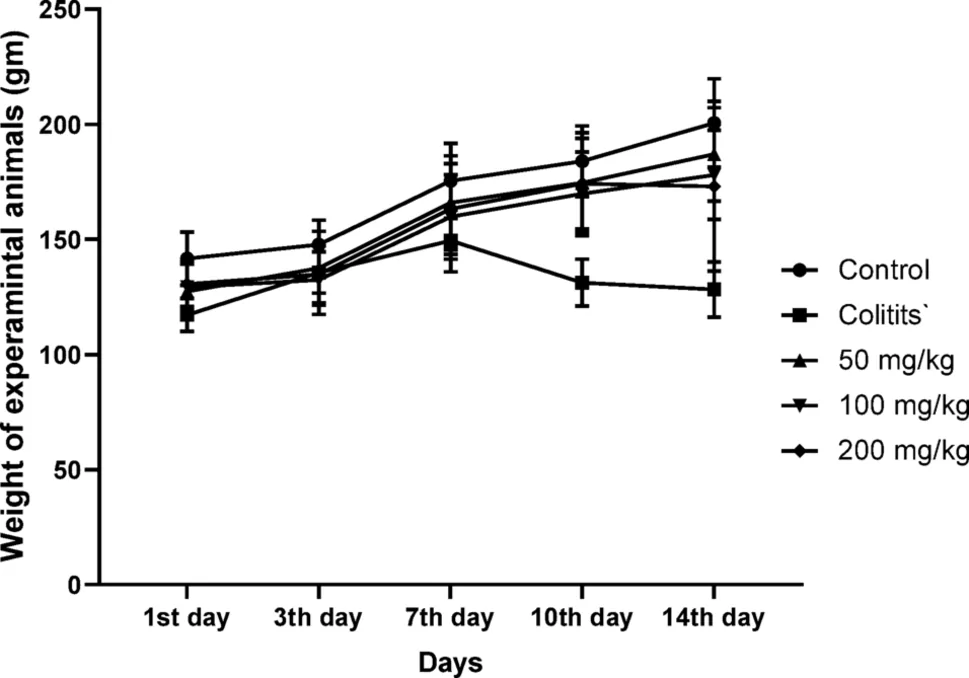
Effect of DPPE on body weight loss in colitis-induced rats. The data are represented as mean ± SD

Body weight was decreased in the colitis-induced group as compared to the control. Otherwise, body weight was increased in all treated groups with DPPE as compared to the colitis‐induced group (Fig. 1).

### Effect of DPPE on blood in stool in colitis-induced rats

**Fig. 2:**
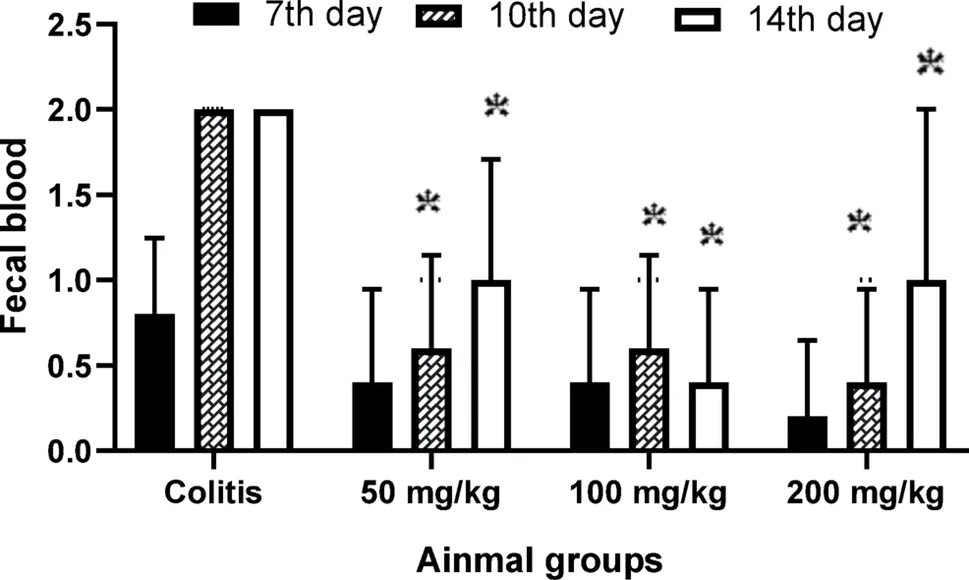
Effect of DPPE on blood in stool in colitis-induced rats. Data is represented as mean ± SD. *Significant at *P* < 0.05 as a comparison colitis-induced group

The significant inhibition was observed in the fecal blood in the treated groups with DPPE (50, 100, 200 mg/kg) on days 10 and 14 as compared to the colitis-induced group (Fig. 2).

### Effect of DPPE on total leukocytes percentage and their differentiation in colitis-induced rats

**Fig. 3:**
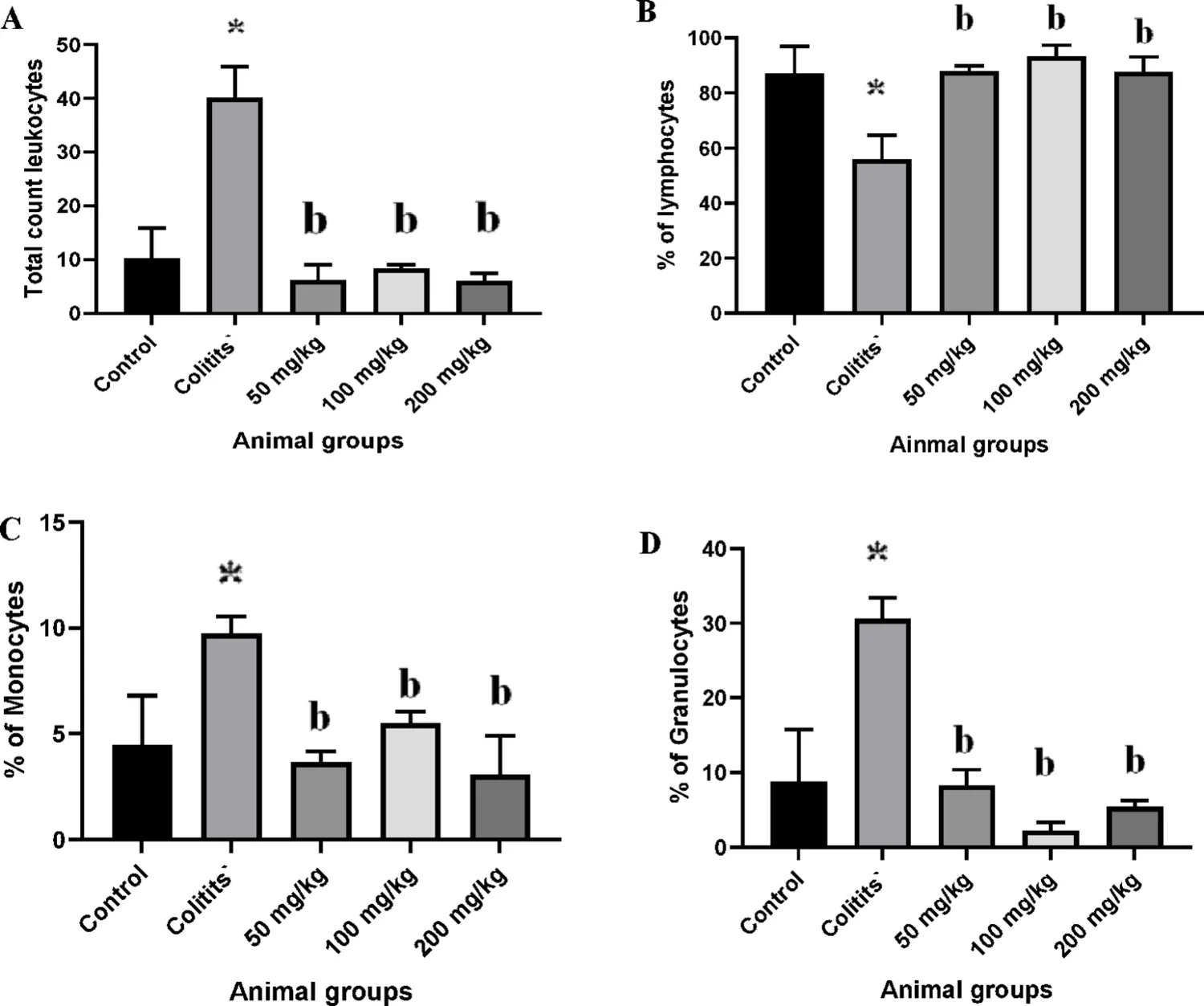
Effect of DPPE on total leukocytes percentage and its differentiation in colitis-induced rats. **A** Total leukocytes count, **B** Lymphocytes (%), **C** Monocytes (%), and **D** Granulocytes (%) in blood of experimental groups. The data are represented as mean ± SD. *Significant at *P* < 0.05 as compared to control. ^b^Significant at *P* < 0.05 as a comparison with colitis-induced group

As shown in Fig. 3, a significant (*P* < 0.05) increase was shown in total leukocyte count in the colitis-induced group as compared to the control, while a significant (*P* < 0.05) decrease in total leukocyte count was reported in treated groups with DPPE (50, 100, and 200 mg/kg) as compared to the colitis-induced group (Fig. 3A). A significant (*P* < 0.05) decrease was shown in the percentage of lymphocytes in the colitis-induced group as compared to the control, while a significant (*P* < 0.05) increase in the percentage of lymphocytes was reported in treated groups with DPPE (50, 100, 200 mg/kg) as compared to the colitis-induced group (Fig. 3B). A significant (*P* < 0.05) enhancement was recorded in monocyte and granulocyte percentages in the colitis-induced group as compared to the control, while a significant (*P* < 0.05) decrease in the percentage of monocytes and granulocytes was reported in treated groups with DPPE (50, 100, and 200 mg/kg) as compared to the colitis-induced group (Fig. 3C, D, respectively).

### Effect of DPPE on histological alterations in colitis

**Fig. 4:**
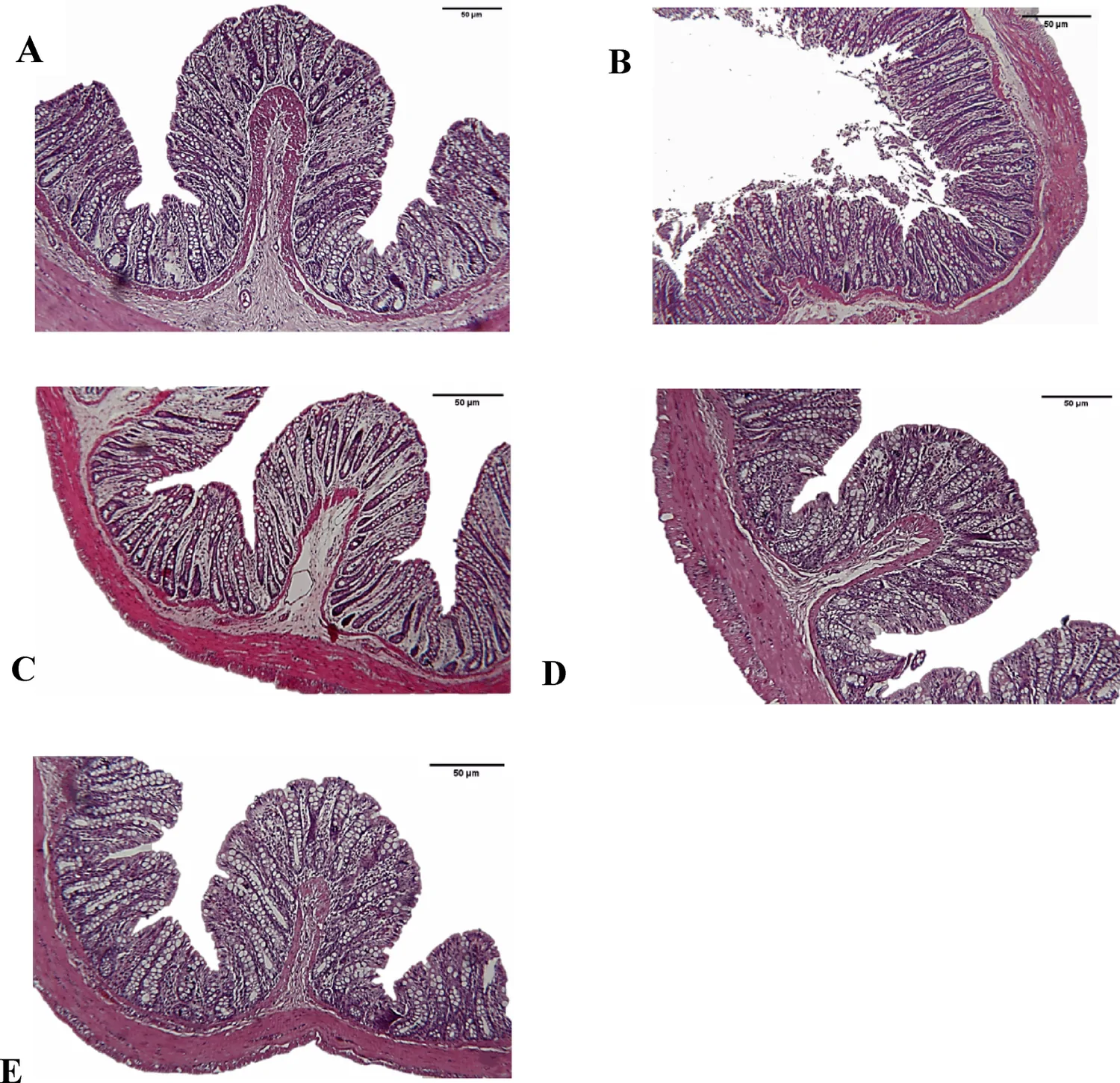
Effect of DPPE on histological alterations in colitis. Images show the staining of rat colon tissue sections with hematoxylin and eosin. **A** Clear intestinal wall structure and regularly arranged microvilli in the normal group. **B** Model rats indicated severe mucosal defects and ulcers. **C**–**E** DPPE-treated rats (50, 100, 200 mg/kg, respectively) demonstrated less mucosal (Scale bar 50 μm)

The histological evaluation of the colonic specimens confirmed the intestinal anti-inflammatory effects exerted by DPPE doses of 50, 100, and 200 mg/kg (Fig. 4C–E, respectively). When compared with normal colonic architecture (Fig. 4A), the colitic control group (Fig. 4B) showed signs of intense transmural inflammation, involving both the mucosa and submucosa layers. Extensive ulceration was also noted, which affected most of the colonic surface and resulted in almost complete destruction of crypts and the absence of goblet cells. The colonic tissue from colitic rats treated with DPPE showed a partial recovery of the mucosa and submucosa, with a lower inflammatory infiltrate, together with the emerging presence of crypts and goblet cells. The beneficial effect of these treatments on colonic architecture was associated with a lower histopathological score in comparison with the colitic control group.

### Effect of DPPE on plasma GST level in colitis-induced rats

**Fig. 5:**
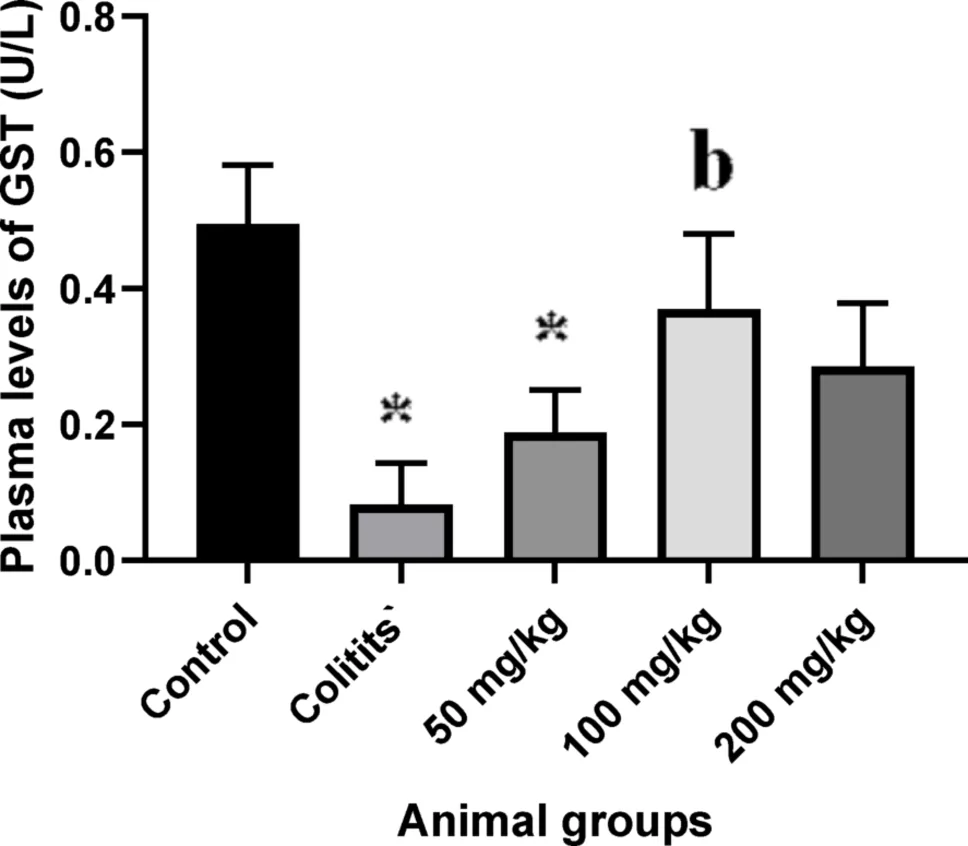
Effect of DPPE on plasma GST level in colitis-induced rats. The data are represented as mean ± SD. *Significant at *P* < 0.05 as compared to control. ^b^Significant at *P* < 0.05 as a comparison colitis-induced group

A significant (*P* > 0.05) decrease in GST level was reported in colitis-induced group and group treated with 50 mg/kg DPPE as compared to control group, while a significant (*P* > 0.05) increase in GST level was recorded in group treated with 100 mg/kg DPPE as compared to the colitis-induced group (Fig. 5).

### Effect of DPPE on plasma H_2_O_2_ level in colitis-induced rats

**Fig. 6:**
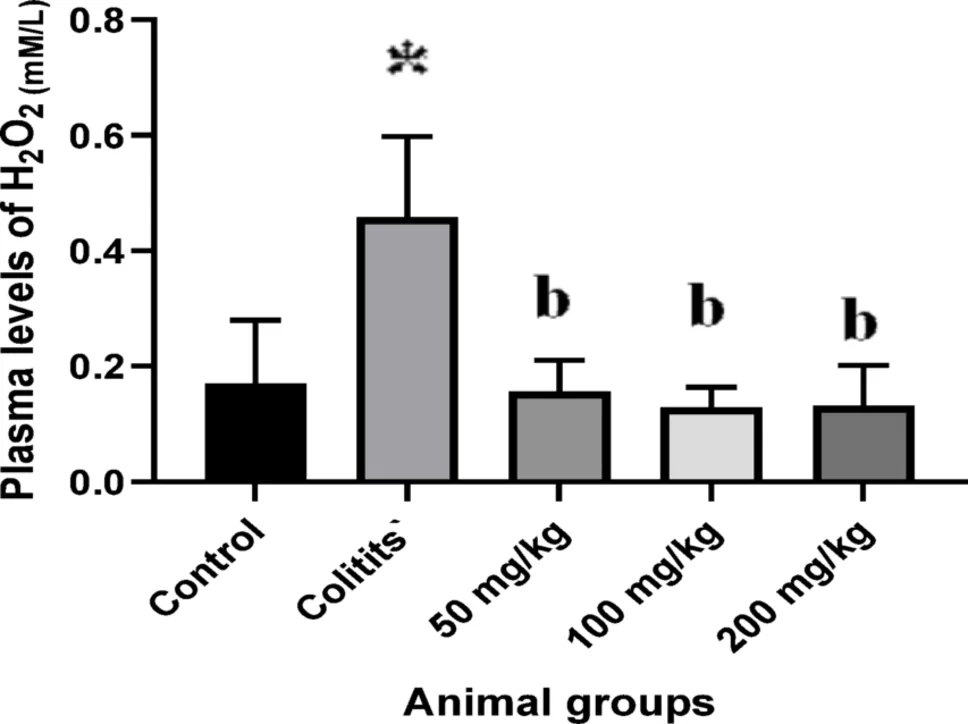
Effect of DPPE on plasma H_2_O_2_ level in colitis-induced rats. The data are represented as mean ± SD. *Significant at *P* < 0.05 as compared to control. ^b^Significant at *P* < 0.05 as a comparison colitis-induced group

A significant (*P* < 0.05) increase in H_2_O_2_ level was reported in the colitis-induced group and treated groups as compared to the control. Otherwise, a significant (*P* < 0.05) decrease in H_2_O_2_ level was reported in all groups treated with DPPE (50, 100, and 200 mg/kg) as compared to colitis-induced groups (Fig. 6).

### Effect of DPPE on intestinal MDA level in colitis-induced rats

**Fig. 7:**
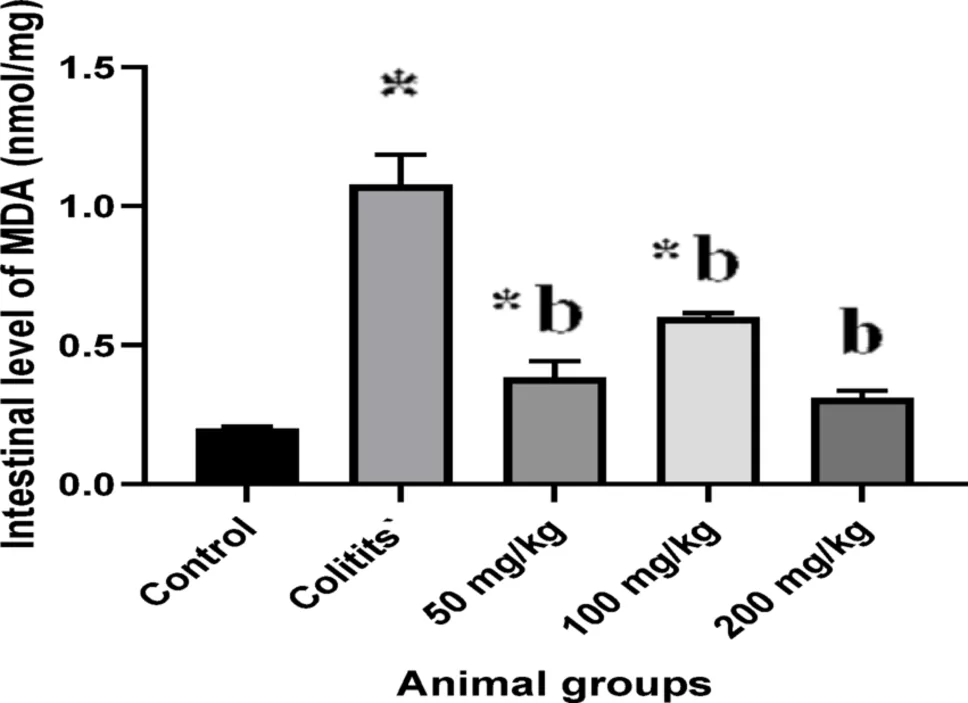
Effect of DPPE on intestinal MDA level in colitis-induced rats. The data are represented as mean ± SD. *Significant at *P* < 0.05 as compared to control. ^b^Significant at *P* < 0.05 as a comparison with colitis-induced group

A significant (*P* < 0.05) increase in intestinal MDA levels was recorded in the colitis group and groups treated with 50 and 100 mg/kg DPPE compared to control, while a significant (*P* < 0.05) decrease in MDA levels was reported in all groups treated with DPPE (50, 100, and 200 mg/kg) as compared to colitis groups (Fig. 7).

### Effect of DPPE on intestinal TNF-α level in colitis-induced rats

**Fig. 8:**
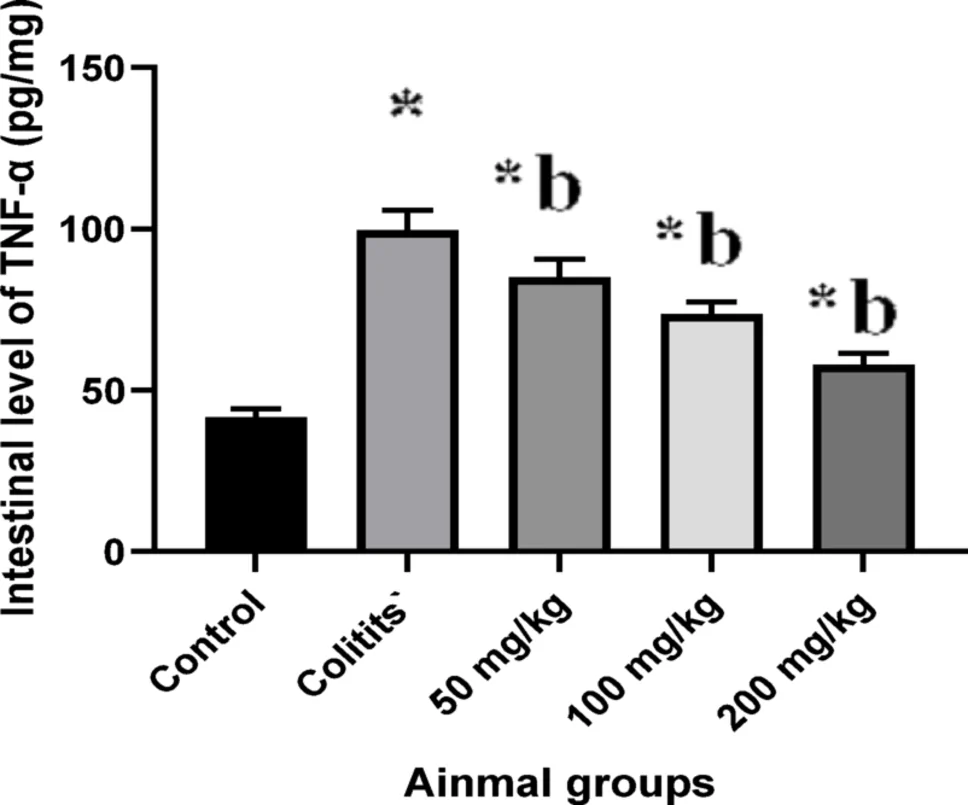
Effect of DPPE on intestinal TNF-α level in colitis-induced rats. The data are represented as mean ± SD. *Significant at *P* < 0.05 as compared to control. ^b^Significant at *P* < 0.05 as a comparison with colitis-induced group

A significant (*P* < 0.05) increase was recorded in intestinal TNF-α level in the colitis group and all treated groups as compared to the control, while a significant (*P* < 0.05) decrease in intestinal TNF-α level was reported in treated groups with DPPE (50, 100, and 200 mg/kg) as compared to the colitis group (Fig. 8).

### Effect of DPPE on intestinal IL-6 level in colitis-induced rats

**Fig. 9:**
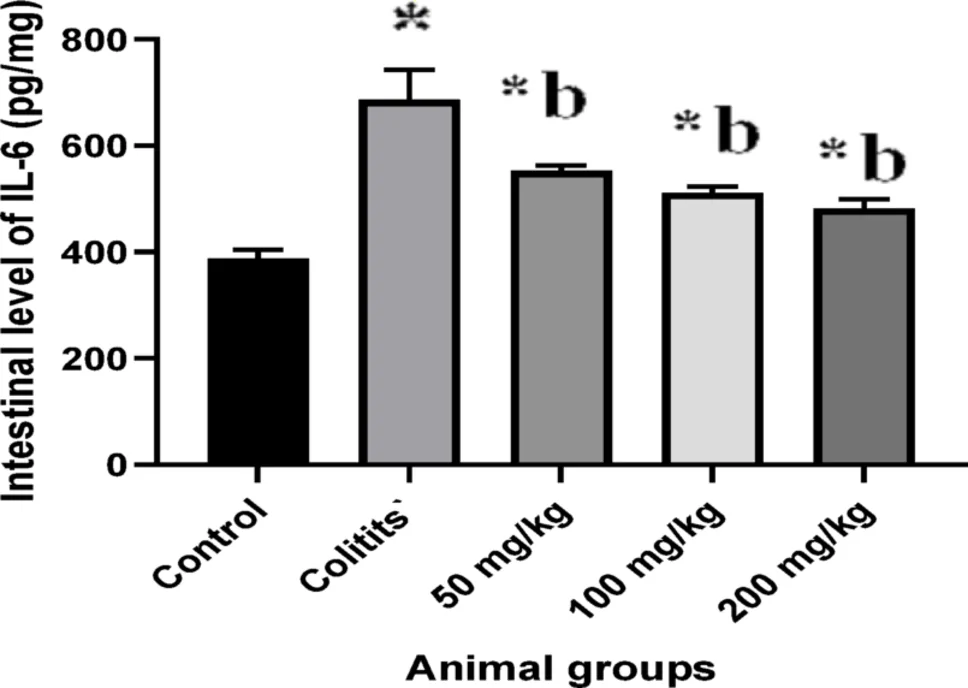
Effect of DPPE on intestinal IL-6 level in colitis-induced rats. The data are represented as mean ± SD. *Significant at *P* < 0.05 as compared to control. ^b^Significant at *P* < 0.05 as a comparison colitis-induced group

A significant (*P* < 0.05) increase was recorded in intestinal IL-6 level in the colitis-induced group and all treated groups as compared to the control, while a significant (*P* < 0.05) decrease in intestinal IL-6 level was reported in treated groups with DPPE (50, 100, and 200 mg/kg) as compared to the colitis-induced group (Fig. 9).

### Effect of DPPE on intestinal IL-1β level in colitis-induced rats

**Fig. 10:**
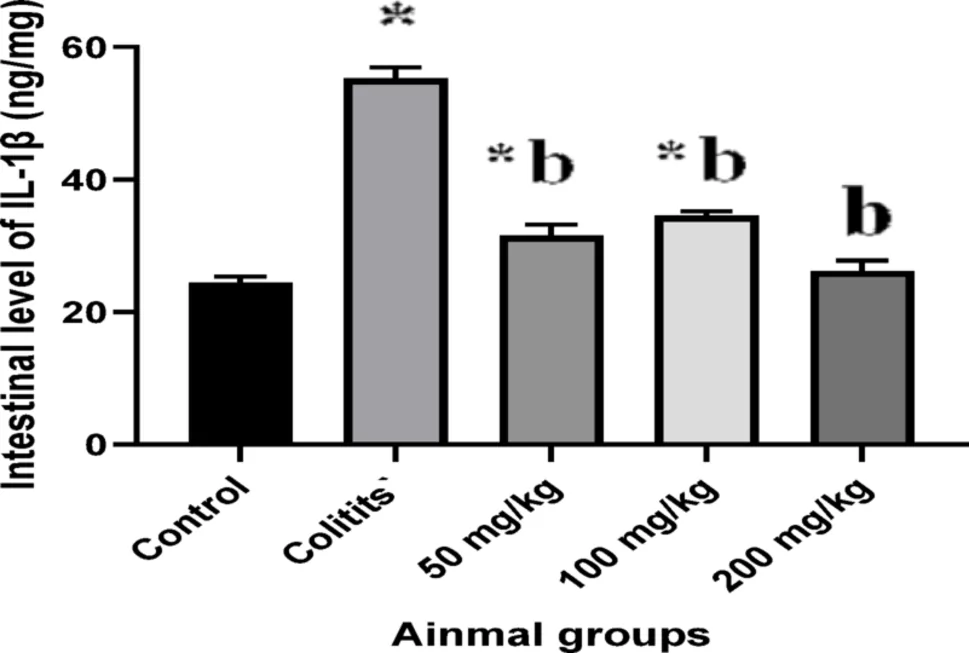
Effect of DPPE on intestinal IL-1β level in colitis-induced rats. The data are represented as mean ± SD. *Significant at *P* < 0.05 as compared to control. ^b^Significant at *P* < 0.05 as a comparison colitis-induced group

Intestinal IL-1β levels were significantly (*P* < 0.05) increased in the colitis group and groups treated with 50 and 100 mg/kg DPPE compared to the control, whereas intestinal IL-1β levels were significantly (*P* < 0.05) decreased in all groups treated with DPPE (50, 100, and 200 mg/kg) compared to colitis groups (Fig. 10).

### Effect of DPPE on intestinal NF-κB level in colitis-induced rats

**Fig. 11:**
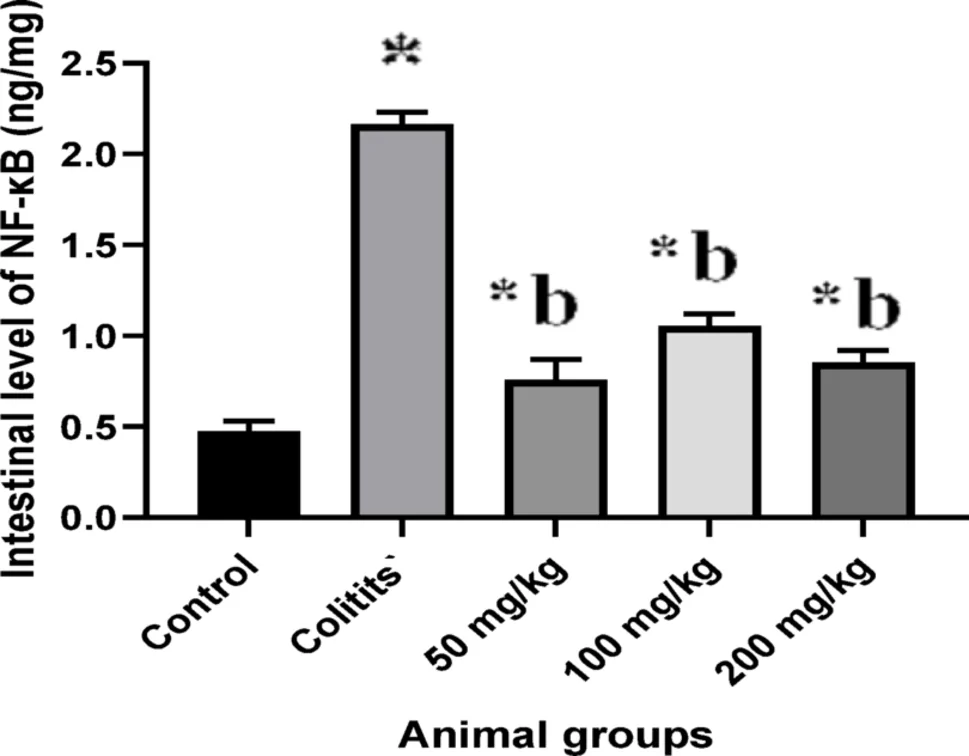
Effect of DPPE on intestinal NF-κB level in colitis-induced rats. The data are represented as mean ± SD. *Significant at *P* < 0.05 as compared to control. ^b^Significant at *P* < 0.05 as a comparison colitis-induced group

A significant (*P* < 0.05) increase was recorded in intestinal NF-κB level in the colitis group and all treated groups as compared to the control, while a significant (*P* < 0.05) decrease in intestinal NF-κB level was reported in treated groups with DPPE (50, 100, and 200 mg/kg) as compared to the colitis group (Fig. 11).

## Discussion

When studying IBD, the best model for investigation is the experimental induction of ulcerative colitis via acetic acid enemas. In this experiment, a 4% concentration of the acid was used, based on a previous study (Özdemi̇R *et al*. 2014). Colonic abnormalities such as inflammation, mucosal ulceration, bleeding, and weight loss are consequences of inflammatory bowel diseases that occur after intrarectal injection of acetic acid in a rat model (Hartmann *et al*. 2012). In addition, due to the injection of acetic acid, white blood cells, such as neutrophils, enter the damaged colon; the colonic barrier breaks down; and inflammatory mediators, such as cytokines and reactive oxygen species, are produced, causing oxidative damage (Ali *et al*. 2017). Date palm pollen (DPP) is noted for its substantial nutritional and pharmacological benefits, enriched with a variety of bioactive phytochemicals and essential nutrients. The chemical profile includes significant amounts of phenolic compounds, flavonoids, fatty acids, phytosterols, proteins, dietary fiber, and necessary minerals, correlating with its antioxidant and biological properties. DPP contains a diverse array of phenolic compounds, with methanolic extracts showing approximately 62.1 mg of total phenolics and 90.2 mg of total flavonoids per gram of extract. High-performance liquid chromatography has detected phenolic acids such as pyrogallol and caffeic acid, alongside flavonoids like quercetin and kaempferol. Apigenin 6-C-glucose-8-C-rhamnose is among the major flavonoids identified, contributing significantly to the antioxidant capabilities of DPP. The lipid content of DPP is characterized by a mix of saturated and unsaturated fatty acids, dominated by palmitic acid, with linoleic (ω-6) and oleic (ω-9) acids as the primary unsaturated types. The nutritional profile improves with ethanol extraction, which enhances unsaturated fatty acid ratios. The unsaponifiable fraction includes phytosterols like β-sitosterol and stigmasterol. (El-Kholy *et al*. 2019; Abdallah *et al*. 2022; Masmoudi *et al*. 2025). DPP is rich in protein (36 g/100 g), carbohydrates (17 g/100 g), and dietary fiber (8 g/100 g), along with essential minerals such as iron, zinc, and magnesium, vital for various biological processes (El-Kholy *et al*. 2019; Abdallah *et al*. 2022; Masmoudi *et al*. 2025).The combinations of its bioactive constituents may provide pharmacological properties of DPP and support its potential application as a natural therapeutic agent for inflammatory disorders, including IBD. During the experimental period, weight loss was observed in the colitis groups, but the control group showed no weight loss (Owusu *et al*. 2020). According to previous studies, colitis causes many symptoms observed in the studied models, such as decreased food in-take, impaired nutrient absorption, and increased metabolic demand during intestinal inflammation. These symptoms are consistent with the findings in this current study. Significant weight gain was observed during the experimental period in the DPPE-treated groups. This improvement is attributed to the rat responding to the treatment, gradually recovering, and regaining weight. The treatment also improved the overall condition of the rats, unlike the control group, which did not experience any weight gain. When examining the blood content in the feces of the rat, no statistically significant differences were observed between the treatments, although the blood content in the feces was lower in the DPPE-treated groups.Changes in blood parameters resulting from tissue damage are among the important clinical manifestations of IBD (Jagtap *et al*. 2011). The current study also demonstrates the immunomodulatory effect of DPPE against inflammation caused by colitis and the alteration in immune cell values. The marked increase in white blood cell, monocyte, and granulocyte counts in patients with ulcerative colitis indicates a sharp rise in systemic inflammation and an enhanced immune response to intestinal injury. By releasing pro-inflammatory cytokines, this increase causes harm to the mucosa. Even though the lymphocyte count was trending downward, this decline was not statistically significant. Consistent with the current findings, earlier investigations have demonstrated lymphocyte suppression in experimental colitis models, which has been linked to stress-induced immunosuppression and potentially T-cell suppression (Olamilosoye *et al*. 2018). The decrease in lymphocytes in this investigation, however, was not statistically significant. Significantly lower white blood cell counts were the outcome of DPPE treatment (at doses of 50, 100, and 200 mg/kg). It’s interesting to note that the significant rise in lymphocytes at the 50 mg/kg dose might be due to an increase in regulatory T cell (Treg) counts, which would suggest a more controlled adaptive immune response, a critical component of tissue repair and long-term recovery. The current study may be compatible with previous findings in mouse models of TNBS-induced colitis, which demonstrated that anti-inflammatory therapies increased regulatory T cells while decreasing pro-inflammatory responses (Zou *et al*. 2015). However, the decrease in lymphocyte count was not statistically significant. White blood cell count in this study was significantly reduced in the DPPE-treated groups at doses of 50, 100, and 200 mg/kg). Lymphocyte count was also significantly increased at the 50 mg/kg dose, possibly due to an increase in Treg. This suggests that adaptive immunity was more responsive and controlled at this dose and played a crucial role in long-term repair and recovery. In addition to the hematological parameters, histological evaluation of colon samples confirmed anti-inflammatory effects in the DPPE-treated groups at the three different concentrations. The colitis group showed severe parietal inflammation, potentially involving the mucosa and submucosa, and also resulted in the absence of goblet cells compared to the normal colonic structure. However, in the treated groups, partial recovery of the mucosa and submucosa was observed, and at the 200 mg/kg dose, colonic tissue regression to near-normal was noted, suggesting that this dose was optimal. Histopathology indicates that the rat responded to the treatment and gradually recovered. Previous observations that may be similar to this study suggest that this increase may be a reaction to compensate for oxidative stress caused by inflammation. Previous observations have shown an increase in glutathione S-alpha (Gsta4) expression in animal models of colitis (Ma *et al*. 2023). GST activity was affected by DPPE treatment in a dose-dependent manner. At a dose of 50 mg/kg, GST activity was significantly reduced compared to both the colitis group and the control group, suggesting a possible inhibitory effect at this dose, which is expected to be suboptimal for response. In contrast, treatment with a 100 mg/kg dose resulted in a significant and statistically significant increase in GST enzyme activity compared to the control group, demonstrating the efficacy of this dose and the rats’ response to treatment in this group. This also suggests optimal enhancement of the colon’s antioxidant defense system. At the highest dose (200 mg/kg), GST enzyme activity decreased again, but without statistical significance, suggesting that these results demonstrate a non-linear response to increasing doses. These results may partially agree with a study on lectins found in bananas, where treatment of TNBS-induced colitis resulted in a slight modification of GST enzyme activity (Miljković *et al*. 2025). All observations indicate that any effective treatment for colitis may enhance antioxidant defenses such as GST enzymes, but suboptimal doses may lead to weak or no responses, thus explaining the dose-dependent effect. In addition to GST enzymes, the current study investigated the effect on H_2_O_2_ levels and how these levels were managed by DPPE. In comparison to the control group, the colitis group’s hydrogen peroxide levels significantly increased as a result of the oxidative stress that occurred in the colon tissues (Strus *et al*. 2009). Treatment with DPPE extract also showed promising results and led to a significant and noticeable decrease in hydrogen peroxide levels compared to the colitis group, demonstrating its effectiveness as an antioxidant and its ability to restore oxidative-reductive balance in colon tissue (Olamilosoye *et al*. 2018). The current study demonstrated a significant decrease in MDA levels after DPPE treatment in comparison to the colitis group, in addition to the inflammatory markers. MDA is regarded as a trustworthy indicator of oxidative stress and lipid peroxidation, indicating the degree of cellular membrane damage caused by a high level of reactive oxygen species. Following DPPE delivery, lower MDA levels were seen, which suggests that DPPE has antioxidant action and improves oxidative state (Hartmann *et al*. 2012). Pro-inflamatory cytokines (TNF-α, IL-6, and IL-1β) play key roles in determining the presence or absence of IBD (Sanchez-Muñoz *et al*. 2008; Guan and Zhang 2017). Acute-phase protein production is stimulated and the complement system is activated, leading to the release of C5 and C3 by IL-6. TNF-α also plays a role in immunity and inflammation by contributing to increased endothelial cell permeability and the formation and activation of leukocytes, which leads to increased prostaglandin production. Both C5 and C3 contribute to neutrophil phagocytosis and leukocyte activation to release more inflammatory mediators and to mast cell activation to release histamine and heparin (Elblehi *et al*. 2021). Additionally, NF-κB expression was significantly reduced after DPPE therapy. One important transcription factor that controls the expression of various inflammatory genes, such as TNF-α, IL-6, and IL-1β, is NF-κB. Consequently, the main mechanism explaining DPPE’s anti-inflammatory actions may be the inhibition of NF-κB activation, which subsequently lowers downstream cytokines. Furthermore, the reduction of MDA observed in the current study may contribute to the attenuation of inflammatory signaling, as oxidative stress is closely associated with activation of the NF-κB pathway and subsequent production of pro-inflammatory cytokines. Therefore, the antioxidant activity of DPP may represent one of the mechanisms responsible for its anti-inflammatory effects (Elblehi* et al*. 2021). These results show that colitis significantly elevates oxidative stress parameters (H_2_O_2_ and MDA) and proinflammatory cytokines (TNF-α, IL-6, and IL-1β) and NF-κB levels in colon tissue, indicating a strong inflammatory response resulting from intestinal inflammation. Furthermore, DPPE treatment significantly reduced the production of these cytokines in all DPPE-treated groups, confirming the anti-inflammatory efficacy of the treatment. The greatest reduction was observed at the maximum dose (200 mg/kg), confirming a dose-dependent effect in inhibiting primary inflammatory mediators, as shown in the results (Elberry *et al*. 2011). Collectively, these findings suggest that DPPE improves inflammatory status and reduces the production of important pro-inflammatory cytokines (TNF-α, IL-6, and IL-1β) via inhibiting the NF-κB-mediated inflammatory pathway. These results provide validity to DPPE’s potential use as a natural treatment for conditions linked to inflammation.

## Limitation of the current study

Despite the promising outcomes of this study, significant limitations exist. Notably, the phytochemical characterization of DPPE was not considered, with biological effects discussed per previous studies on the same species. In addition, this study lacks the presence of a comparison between the optimized DPPE concentration and currently available standard therapies. Future investigations are warranted to compare DPPE with conventional treatments to determine its relative efficacy and potential clinical relevance. Additionally, the study’s parameters did not cover inflammatory mechanisms, indicating that future investigations will explore further molecular pathways and mediators for documenting a DPPE’s protective benefits.

## Conclusions

In this study, DPPE showed a protective effect in acetic acid–induced colitis in rats. DPPE administration significantly reduced oxidative stress and attenuated pro-inflammatory cytokine expression. Additionally, histopathological examination revealed noticeable improvement in colonic tissue architecture compared to untreated colitis groups.

**Fig. 12:**
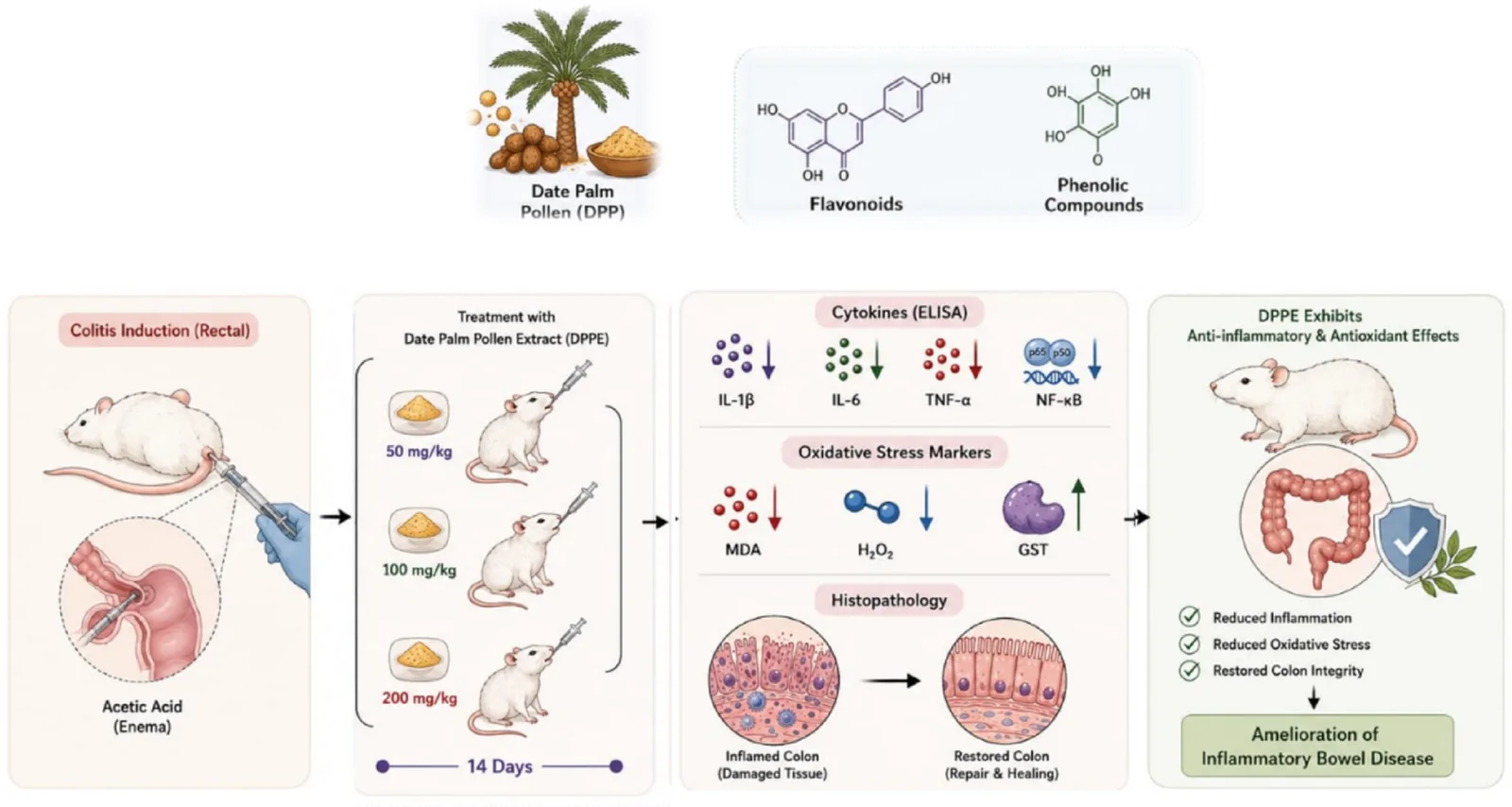
Diagrammatic summary of the proposed mechanisms of how date palm pollen extract ameliorates inflammatory bowel disease

These findings suggest that DPPE may represent a promising natural anti-inflammatory candidate in experimental colitis. In addition, the current results supported using date palm pollen in enhancement treatment of IBD, especially given the side effects of conventional drug therapies (Fig. 12).

## Data Availability

The original contributions presented in this study are included in the article/supplementary material. Further inquiries can be directed to the corresponding author.

## Abbreviations

IBD: Inflammatory bowel disease
DPPE: Date palm pollen extract
